# Improved home management of oral pediatric anticancer drugs as a result of an intervention comprising practical training, written instructions and movie clips: A pilot study

**DOI:** 10.1177/10781552221080445

**Published:** 2022-02-14

**Authors:** Ranaa Akkawi El Edelbi, Staffan Eksborg, Jennie Ekman, Synnöve Lindemalm

**Affiliations:** 1 Department of Women's and Children's Health, Childhood Cancer Research Unit, 27106 Karolinska Institutet, Stockholm, Sweden; 2 Division of Pediatrics, 59562 Karolinska University Hospital, Astrid Lindgren Children's Hospital, Stockholm, Sweden; 3 Department of Clinical Sciences, 27106 Karolinska Institutet, Intervention and Technology (CLINTEC), Stockholm, Sweden

**Keywords:** Oral anticancer drugs, handling, home setting, pediatric, intervention

## Abstract

**Background:**

Long term treatment of pediatric patients with oral anticancer drugs (OADs) requires the parents/caregivers to prepare the drug at home. The handling procedures in the home setting are, however, not regulated by Swedish law and the parents are often left without guidance on how to handle OADs in a safe way.

**Aim:**

The aim of this study was to increase understanding of how OADs are handled by parents/caregivers in the home setting before and after an intervention.

**Methods:**

Parents of pediatric cancer patients were observed and videotaped during their handling of OADs in the home setting before and after the intervention. During the intervention, the parents were provided with written instructions, movie clips and practical training on handling the OADs. Four checklists were used to compare and score the four handling procedures (measuring an oral suspension, cutting tablets, dissolving tablets, and opening capsules) for each parent before and after the intervention.

**Results:**

The intervention significantly improved the OAD handling procedures among the studied parents. The median score for correct handling was 19% (IQR: 3.6 to 30%) before the intervention and 89.5% (IQR: 71.5 to 94.5%) after the intervention (p < 0.0001).

**Conclusions:**

An intervention comprising practical training and information presented in different forms improved the handling of OADs at home by parents. There is an urgent need to implement this method in all oncology centers in Sweden, educate HCPs to standardize the presentation of information. There is also a great need to provide parents with age-appropriate oral drug formulations from the local hospital pharmacies in Sweden.

## Introduction

Long term treatment with oral anticancer drugs (OADs) is very common today in many pediatric oncology therapy protocols; these protocols enable the patients or their parents/caregivers to prepare and administer the drugs at home. OADs offer many advantages, such as convenience for the patient and parents and the absence of need for intravenous access, with consequent improved quality of life.^1–5^ However, problems associated with the shift of responsibility from a controlled healthcare setting to the home environment have been identified. The Swedish Work Environment Authority has regulated the handling of cytotoxic agents to protect Healthcare Professionals (HCPs) and pharmacists from unintended occupational exposure to these agents. All reconstitution and handling should be carried out at a pharmacy, not in the ward, to avoid drug toxicity for HCPs. However, there are no regulations in Sweden to control the handling and manipulation of OADs in the home setting. There are also no age-appropriate formulations of OADs that could provide flexible doses to suit children of all ages.^6^ Not all OADs are available in liquid oral forms and, consequently, parents need to prepare the solid drugs for administration at home. The manipulation processes are complex, which can increase the risk of improper drug handling^7–10^ and affect the accuracy of the dose, along with the stability and bioavailability of the drug.^6,11^

Parents, and also HCPs in some cases, are often unaware of the risks associated with handling OADs and the bodily excretions of patients receiving them and can believe that OADs are not as toxic and potent as their intravenous counterparts.^2,4,12^ Preparation of parenteral chemotherapy agents is associated with a high risk of accidental exposure through dermal contact with the drugs. The health effects associated with this exposure include both carcinogenic and reproductive outcomes.^12–14^ The risks associated with handling OADs are still unknown but skin contact with OADs during drug handling or handling of bodily excretions has been linked with the discovery of drug residues in the urine.^15^ Wearing gloves and washing hands are essential when preparing and administering OADs.^15,16^ Parents also risk exposure from dust particles from drugs released into the air and inhaled, e.g. when opening a capsule, from contaminated surfaces, or when re-using protective personal equipment (PPE).^2,17,18^ There are no safe levels for hazardous drug exposure. Inappropriate handling of OADs and the patient's bodily excretions poses significant healthcare risks which can compromise patient safety and contribute to increases in medication errors, unintended exposure for family members, contamination of household surfaces and possible pollution of the water supply and landfills.^1,18–20^ There is a need to educate parents/patients through practical training and to supply them with educational materials in different forms and languages in order to optimize the handling of OADs and bodily excretions.^12,21^

Historically, Swedish hospital pharmacies have compounded suitable child-friendly formulae from solid anticancer formulations (e.g. tablets or capsules) in order to provide an accurate dose, facilitate drug intake and reduce the handling of OADs for nurses and parents. However, the compounding of oral anticancer formulae in pharmacies was discontinued in 2009 in order to minimize the risk of exposure for the pharmacy employees. Today, the parents of pediatric cancer patients are responsible for manipulating and handling the drugs in the home setting.

The pharmacist working group in the Nordic Society Of Pediatric Hematology and Oncology (NOPHO), in cooperation with Experienced and Evidence-based Pediatric Drugs in Sweden (ePed),^22^ convened a meeting on this subject in Dec 2019 and established standards providing information on best practice for giving OADs to pediatric patients in the home setting. Pharmacists in the Nordic countries of Finland, Norway, Sweden, Latvia and Estonia identified strategies for handling OADs in a safe and optimal way at home. The group reached consensus on: a) how to split tablets, b) how to handle oral suspensions/solutions, c) how to open capsules, providing two different methods, and d) how to dissolve tablets. The pharmacists cooperated with nurses working locally in pediatric oncology clinics. The strategies were based on international guidelines, studies and best practice methodology for the handling of oral cytotoxic drugs.^2,16,18,19,23–28^

The aim of this study was to investigate any changes in the handling procedures for OADs in a home setting before and after an intervention, and to compare these with the best practice standard.

## Methods

The term ‘manipulation’ refers in this study to cutting or dissolving tablets and opening capsules to access the contents.

1) Study subjects

The parents of all patients in a pediatric oncology center in Sweden during the study period were candidates for participation in both parts of the study (Part 1 before the intervention and Part 2 where parents received the intervention and were then observed). The parents were recruited by telephone from the pediatric oncology center. The participants were selected using purposeful sampling which took into consideration the OAD formulation that the child was taking, the manipulation method required, and the duration of the treatment period. We excluded parents who did not have to manipulate the drug, were not able to communicate in Swedish without being provided with an interpreter, or could not be reached by public transport.

The study was a pilot study and no statistical power calculation was provided.

2) Materials

The best practice standard contained information on the production of:

- Supportive guidelines for parents/patients with written instructions and movie clips on handling procedures for OADs at home.
- Supportive guidelines for standardizing the information given by HCPs for educating the parents of pediatric patients on how to handle OADs at home.

a) Patient education material included:

- Caregiver's education
- Practical training

b) Scoring tools

Four checklists were used to score the handling procedures for OADs (measuring an oral suspension, cutting tablets, dissolving tablets, and opening capsules) as recommended by the NOPHO pharmacist group and ePed.se, Table 1. The checklists were used to score the handling procedures by each parent individually.

**Table 1.** Results observations - preparation of oral chemotherapy.

	**Before**	**After**
	**intervention**	**intervention**
	**Responses (%)**	**Responses (%)**
**General procedures**		
Washed hands with soap and water before beginning to prepare the drug	17%	79%
Used new gloves	22%	93%
Reused gloves	6%	0%
Placed protective cover over working area	17%	93%
Disposed used material in a sealed bag, in the regular garbage	0%	93%
Cleaned working area with household cleaners and water	0%	57%
Washed hands with soap and water after completing the preparation of the drug	28%	71%
**Oral suspension**		
Used adapter to measure the prescribed dose	67%	100%
Used new oral syringes	33%	80%
Reused oral syringes	67%	20%
**Dissolving tablets**		
Used new oral syringes/medical cups	67%	100%
Reused oral syringes/medical cups	33%	0%
Syringe cork	33%	67%
Washed spoon and glass cup with detergent and water, let airdry	0%	50%
**Cutting tablets**		
Used tablet cutter	50%	100%
Cleaned tablet cutter with detergent and water, let airdry	17%	100%
**Opening capsules**		
Used new oral syringes	0%	100%
Used new medicine cups	0%	100%
Reused oral syringes	50%	0%
Washed spoon and glass cup with detergent and water, let airdry	0%	0%

3) Observation and intervention

a) Part 1 (before intervention)

Initially, parents of pediatric oncology patients received standard information orally from nurses and/or doctors on the handling of OADs, as provided in routine care at the hospital.

Home observations were conducted between October 15 and November 15 2019, when a pharmacist visited the parents in their homes to observe their handling of OADs. Preparation of the OADs by the parents was videotaped.

b) Intervention: training and education

### Caregivers’ education

Six to seven months after the first observations were made, parents of pediatric oncology patients were again recruited to participate in the intervention. They were provided with tools to facilitate the handling of OADs at home (starting kit) and a written instruction sheet that included illustrations on how to handle the drug at home; these were to be studied before the training session.

### Practical training

A pediatric nurse then trained the parents on how to handle the OADs at home via a communication tool for video calls (Skype), and the sessions were recorded.^29^ The nurse focused the training session on correct handling techniques, use of personal protective equipment, cleaning of the working area and safe disposal of the remains. The parents, with the starting kit available, were then asked to demonstrate the handling procedures for OADs, and were given the opportunity to ask questions after the session. They were provided with information on how to handle bodily excretions and how to check ePed.se to find information on drug handling presented in different forms (written instructions and video clips) and other aspects of using OADs.

Ten to 18 days after the intervention, the handling procedures for OADs were again recorded, this time via Skype. These observations were carried out between May and July 2020.

c) Part 2 (after intervention)

Each parent received a score of either 0 (incorrect/mishandled) or 1 (correct) for each step of the procedure. The maximum possible score (i.e. 100%) were 19 for handling oral suspensions, 18 for cutting tablets, 21 for dissolving tablets and 24 for opening capsules.

### Ethical considerations

The study was approved by the Regional Ethical Review Board in Stockholm (DNR: 2019–03192). Oral and written information about the purpose of the study, confidentiality and the right to discontinue their participation at any time was provided to all participants. Participants were also asked to sign a consent form before the beginning of the observation and videotaping sessions. For those observations conducted through Skype, consent was given orally, and was audio-recorded.

### Statistical analysis

Mann-Whitney U-tests were used for comparing the two independent populations (Parts 1 and 2 of the study)**.** P-values < 0.05 were considered statistically significant. Reported p-values are from two-sided tests.

## Results

### Parent characteristics

**Figure 1.**
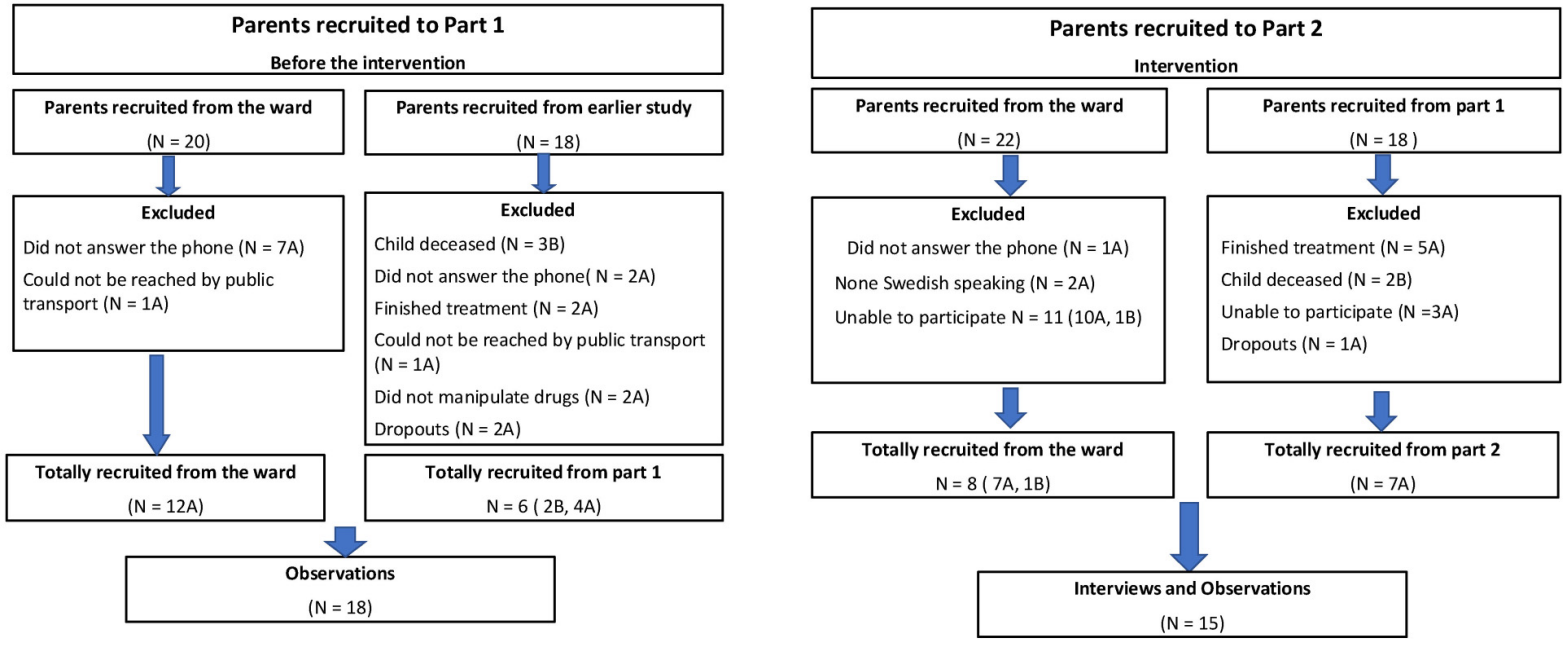
CONSORT flow diagram. Summary of the total number of parents of children with cancer recruited to (a) Part 1 of the study (where they were observed without the intervention), and (b) Part 2 of the study (where they received the intervention and were then observed).

The participants were parents of pediatric patients who had acute lymphoblastic leukemia (ALL) or brain tumors and who were treated with OADs at home. Eighteen parents were included in Part 1 (Figure 1(a)) and 15 parents were included in Part 2 (Figure 1(b)). Six parents in Part 1 had already participated in an earlier similar study. Seven parents in Part 2 had also participated in Part 1 of the study. Demographic data are presented in Table 2. Eighty-three percent of the parents in Part 1 and 87% of those recruited for Part 2 were of female gender. Twenty-eight percent and 67% of the parents in Parts 1 and 2 of the study had been handling the drug for more than one year. Seventy-two percent and 33% of the parents in Parts 1 and 2 had been handling the drug for less than or equal to one year. In both parts of the study, 28% of the parents had a non-Swedish background, Table 2.

A = acute lymphoblastic leukemia, B = brain tumor.

**Table 2.** Demographic data of the child and participating parents.

	**Part 1**	**Part 2**
	**No intervention**	**Intervention**
Age (years)	5 (2–10)	5 (1–18)
Patients	18	15
Female gender		
Parents	18	15
Female gender	83% (15)	87% (13)
Treatment with oral anticancer drugs < 1 year	72% (13)	33% (5)
Treatment with oral anticancer drugs > 1 year	28% (5)	67% (10)
Diagnosis		
Leukemia	89% (16)	93% (14)
Brain tumor	11% (2)	7% (1)
Parents with non-Swedish background	28% (5)	28% (5)
Data are expressed as median (range) or percent (N)		

### Before the intervention

#### General procedures

Seventeen percent of the parents washed their hands with soap and water before preparing the drug. Only 22% of the parents used new gloves, 6% re-used gloves and 17% placed protective covering over the working area. None of the parents disposed of the used equipment in a sealed bag in the regular garbage, or cleaned the working area after finishing drug preparation. Only 28% washed their hands with soap and water after completing drug preparation, Table 1.

#### Oral suspension

Thirty-nine percent of the parents (7 of 18) had to measure the prescribed dose of oral suspension; of those, 67% used an adapter, 33% used new syringes and 67% re-used oral syringes, Table 1.

#### Dissolving tablets

Seventeen percent of the parents (3 of 18) had to dissolve tablets in water during the preparation phase. Sixty-seven percent used new oral syringes/medicine cups, 33% re-used oral syringes/medicine cups, and 33% used an oral syringe with a cork. None of the parents washed the used spoon and glass cup with detergent and water, Table 1.

#### Cutting tablets

Thirty-three percent of the parents (6 of 18) had to cut the tablets to deliver the prescribed dose; of those, 50% used a tablet cutter. Only 17% cleaned the tablet cutter with detergent and water after use, Table 1.

#### Opening capsules

Eleven percent of the parents (2 of 18) had to open a capsule and give half of the contents to their child. Of those, neither used a new oral syringe or medicine cup and one (50%) re-used an oral syringe. Neither cleaned the spoon and glass cup with detergent and water after use, Table 1.

### After the intervention

#### General procedures

After the intervention, 79% of the parents washed their hands with soap and water before preparing the drug, and 93% used new gloves and protective covering and disposed of the used equipment in a sealed bag in the regular garbage. Only 57% cleaned the working area and 71% washed their hands with soap and water after completing the drug preparation, Table 1.

#### Oral suspension

Thirty-three percent of the parents (5 of 15) prepared an oral suspension; of those, all five (100%) used an adapter, 80% used new syringes during the preparation and 20% re-used oral syringes, Table 1.

#### Dissolving tablets

Forty percent of the parents (6 of 15) were instructed to dissolve tablets when preparing the drug. All six used new syringes but only 67% used a syringe cork. Only 50% washed the used spoon and glass cup with detergent and water, Table 1.

#### Cutting tablets

Twenty percent of the parents (3 of 15) had to cut the tablets during drug preparation; all three used a tablet cutter and cleaned the tablet cutter with detergent and water after use, Table 1.

#### Opening capsules

Seven percent of the parents (one of 15) had to open a capsule to give one quarter of the content to the child. The parent used new syringes and medicine cups but did not clean the spoon and glass cup with detergent and water after use, Table 1.

### Handling of OADs before and after caregiver education

The handling score was composed of the total individual score for each participant as a percentage of the maximum possible score for the relevant method.

**Figure 2.**
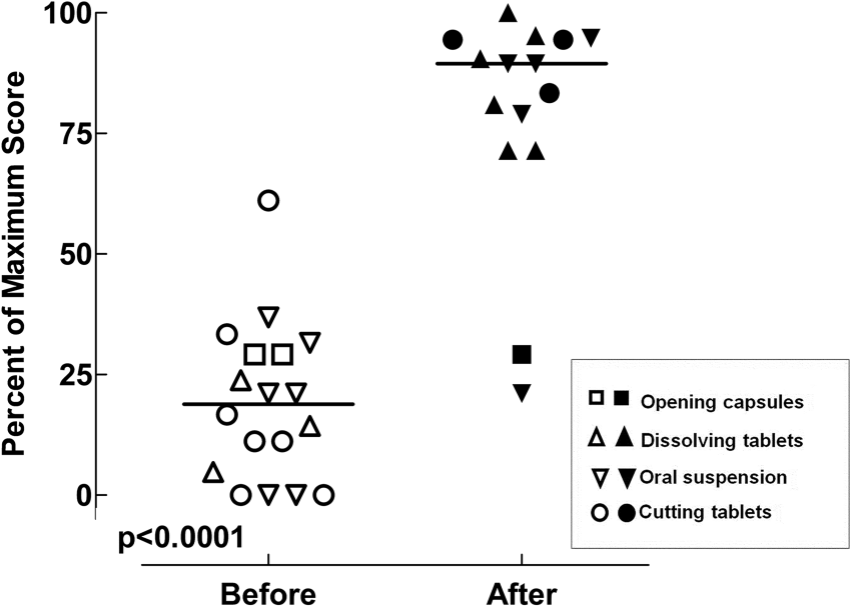
Handling procedures for oral anticancer drugs before and after the intervention. The figure shows the changes in the handling scores (as a percentage of the maximum scores for each handling procedure) before and after the intervention. The unfilled symbols represent scores before the intervention; the filled symbols represent scores after the intervention.

Before the intervention, the median handling score for all the included methods was 19% (IQR: 3.6 to 30%), compared to 89.5% (IQR: 71.5 to 94.5%) after the intervention (significantly higher; p < 0.0001), Figure 2.

## Discussion

Long term treatment with OADs is common in many pediatric oncology protocols.^12,30^ Guidelines on the safe handling of OADs for HCPs have been internationally available since 1985; e.g. the National Institute for Occupational Safety Health (NIOSH)^28^ and Oncology Nursing Society (ONS)^31^ guidelines. However, precautions for handling OADs at home have not been properly incorporated into these guidelines. At our hospital there are currently no standard procedures for how to inform parents on safe handling of OADs at home. Nor are parents at our pediatric oncology clinic provided with suitable materials for carrying out the procedures, e.g. gloves and oral syringes.

The results of this pilot study have confirmed that parents are not handling OADs correctly or safely at home without intervention. The intervention, comprising practical training, written instructions and movie clips, significantly increased the incidence of correct safe handling of OADs. These results are similar to those of previous studies where pharmaceutical counseling significantly decreased deficits in knowledge about OAD management among parents, from 24% to 8%.^1^ In contrast, we observed handling of OADs at home “in real life”, both before and after the intervention. We also educated parents on the handling procedures and provided them with materials in different forms.

Providing an intervention to the parents of children with brain tumors who need to manipulate capsules is urgent. The handling technique is complicated, capsules give rise to more dust than tablets, and these children are often more severely affected by their disease than those with ALL.

During the study, we observed that it was mostly mothers who had the care of the child's medications at home. That is true for most ALL patients, but not for those handling thalidomide capsules, which are mostly given to pediatric patients with brain tumors. The mothers in those cases were carefully instructed not to handle the thalidomide capsules as they could be pregnant and thalidomide being a confirmed teratogen, but it is, however, also assumed that male fertility is affected by thalidomide.^32,33^

We included parents of children with non-Swedish-speaking backgrounds. To be able to fully understand the handling procedure and facilitate drug handling we believe it is important to provide those parents with materials in their mother tongue.

All observations after the intervention were conducted via Skype because of the Covid-19 pandemic. Having the opportunity to observe the parents via Skype was convenient for the study team and for most parents, since they could be more flexible when choosing the observation date and time. It also meant that, despite some of the observations being conducted during the summer holidays, parents could easily join us from their summer houses if necessary. However, we were not able to observe the whole handling process (e.g. storage of the drugs or the process of drug intake) by this method. A few parents also had some technical issues using Skype.

The observed handling practices among the parents before the intervention showed variability in using personal protective equipment (e.g. gloves), re-use of oral syringes, unsafe disposal habits and proper handling techniques. These results are in line with a study where only 10% of the parents used gloves, 30% placed a protective covering over the working area, 1% did not re-use oral syringes, and 50% used a tablet cutter.^10^

Handling the drugs in an unsafe and incorrect way at home exposes not only the handler but all family members to cytotoxic drugs. There is an increased practice of caring for patients as outpatients, lack of drug formulations customized for children, and lack of an organized way of providing parents with suitable materials (e.g. gloves). Consequently there is a need to educate parents in drug management at home, especially those handling complex processes and management of high-risk and cytotoxic medicines such as OADs.^29^

It is assumed that errors in managing medicines are the result of lack of knowledge and that patient/parent education is crucial.^1,30,34^ During the intervention we combined education in different forms, not only written and video clips but also practical training. Combining different educational forms gives the best outcomes for successful OAD treatment.^2,5,30^ Patient/parent training not only minimizes exposure to OADs but also promotes patient safety, and optimizes dosing, adherence to the treatment and knowledge about important side effects.^5^

In order to increase patient safety, there is a need to implement the guidelines on safe handling of OADs at our hospital, to educate nurses and physicians, and to standardize the information given to parents and patients. The handling procedures for OADs were significantly improved after the intervention but there seems to still be a need to repeat the information and to support parents in handling procedures during the treatment period. Studies have confirmed that patients’ and parents’ handling and disposal of OADs are inconsistent and that they often do not follow the published recommendations.^2^

We believe that it is ethically unacceptable to expect that parents should professionally handle OADs at home on their own. There is an urgent need to provide parents with oral suspensions that have been reconstituted by a pharmacy as is currently occurring in, for example, Finland.

### Limitations

Our study had a number of limitations, as follows. Firstly, the study had only a small sample size, lacked parents with newly diagnosed children, and only one pediatric oncology center was included. Our conclusions cannot, therefore, be generalized to other oncology centers. However, we know that no oncology centers in Sweden combine different forms of information with practical training for parents. Another limitation is that we only included a few drugs, although all manipulation procedures were included. We should also consider the Hawthorn effect,^35^ where many parents may have handled the drugs correctly after the intervention only because they were observed. Because of the Covid-19 pandemic after the intervention, all observations were conducted via Skype. Because we were not able to visit the parents in their homes, we could not observe the handling of OADs in the correct context. There is consequently a need to perform large multicenter studies over a longer period and including parents of newly diagnosed children.

### Strengths

The strengths of this study that should be considered are as follows. We visited the parents in their homes where they usually handled the OADs and observed the handling procedures before the intervention. What we observed in the homes, including the risks associated with the handling procedures, helped us to optimize the consensus guidelines and to focus on risk-reducing strategies. The combination of different forms for delivering the information, including practical training, is believed to result in better outcomes because of a synergistic effect, according to a Cochrane review.^36^

Having parents observed via Skype also had advantages: the parents felt more flexible because they could adjust the Skype meetings to their daily life routines.

## Conclusions

An intervention combining practical training and information presented in different forms supported the parents in handling OADs in an optimal way at home. There is an urgent need to implement this method in all oncology centers in Sweden, educate HCPs to standardize the presentation of information. There is also a great need to provide the parents with age-appropriate oral drug formulations from the local hospital pharmacies in Sweden.
